# Evolutionarily Conserved Transcriptional Co-Expression Guiding Embryonic Stem Cell Differentiation

**DOI:** 10.1371/journal.pone.0003406

**Published:** 2008-10-15

**Authors:** Yu Sun, Huai Li, Ying Liu, Mark P. Mattson, Mahendra S. Rao, Ming Zhan

**Affiliations:** 1 Bioinformatics Unit, Research Resources Branch, National Institute on Aging, National Institutes of Health, Baltimore, Maryland, United States of America; 2 The CRL, Invitrogen Corporation, Carlsbad, California, United States of America; 3 Laboratory of Neurosciences, National Institute on Aging, National Institutes of Health, Baltimore, Maryland, United States of America; The Research Institute for Children at Children's Hospital New Orleans, United States of America

## Abstract

**Background:**

Understanding the molecular mechanisms controlling pluripotency in embryonic stem cells (ESCs) is of central importance towards realizing their potentials in medicine and science. Cross-species examination of transcriptional co-expression allows elucidation of fundamental and species-specific mechanisms regulating ESC self-renewal or differentiation.

**Methodology/Principal Findings:**

We examined transcriptional co-expression of ESCs from pathways to global networks under the framework of human-mouse comparisons. Using generalized singular value decomposition and comparative partition around medoids algorithms, evolutionarily conserved and divergent transcriptional co-expression regulating pluripotency were identified from ESC-critical pathways including ACTIVIN/NODAL, ATK/PTEN, BMP, CELL CYCLE, JAK/STAT, PI3K, TGFβ and WNT. A set of transcription factors, including FOX, GATA, MYB, NANOG, OCT, PAX, SOX and STAT, and the FGF response element were identified that represent key regulators underlying the transcriptional co-expression. By transcriptional intervention conducted *in silico*, dynamic behavior of pathways was examined, which demonstrate how much and in which specific ways each gene or gene combination effects the behavior transition of a pathway in response to ESC differentiation or pluripotency induction. The global co-expression networks of ESCs were dominated by highly connected hub genes such as IGF2, JARID2, LCK, MYCN, NASP, OCT4, ORC1L, PHC1 and RUVBL1, which are possibly critical in determining the fate of ESCs.

**Conclusions/Significance:**

Through these studies, evolutionary conservation at genomic, transcriptomic, and network levels is shown to be an effective predictor of molecular factors and mechanisms controlling ESC development. Various hypotheses regarding mechanisms controlling ESC development were generated, which could be further validated by *in vitro* experiments. Our findings shed light on the systems-level understanding of how ESC differentiation or pluripotency arises from the connectivity or networks of genes, and provide a “road-map” for further experimental investigation.

## Introduction

Embryonic stem cells (ESCs) are pluripotent; they can replicate indefinitely and differentiate into multiple tissues from all three embryonic germ layers. Due to their unique properties, ESCs serve as a model system for studying embryo development and hold great promise for regenerative medicine [1], [2]. An understanding of the molecular mechanisms regulating pluripotency of ESCs is critical in realizing their therapeutic and biological potentials. Previous studies examining differentially expressed genes in ESCs and their early-differentiated counterparts, embryoid bodies (EBs), have begun to identify the molecular signatures of ESCs and elucidate the mechanisms controlling pluripotency [3]–[7]. Yet, significant differences exist among ESCs harvested from different species [8]–[10], suggesting that cross-species analysis may help distinguish between fundamental and species-specific mechanisms regulating ESC development. We previously conducted a human-mouse comparative genomics study on pathways critical for ESC self-renewal and differentiation [11]. The study demonstrates that the pathways directed by FGF, NANOG, NODAL, OCT4 and SOX2 are evolutionarily conserved as the component genes are conserved on both the gene and promoter structure. The LIF pathway is, on the other hand, evolutionarily divergent from the genomic perspectives. The study suggests that the conserved OCT4/SOX2 synergistic action is an important activation mechanism in the FGF, LIF, NANOG and OCT4 directed pathways, which are furthermore regulated by a feedback loop formed by ESG1, FOXD3 and SOX2. FGF may regulate ESG1, FOXD3 and SOX2 in a parallel pathway to maintain ESC self-renewal. We have also conducted a comparative transcriptomics study on ESCs, which examined a large set of biological pathways and processes, as well as transcription factors and growth factors expressed in ESCs [12]. In that study, embryo development, pattern specification, cell cycle, apoptosis, NOTCH, NODAL and other pathways were found to be transcriptionally conserved as the genes of these pathways show a significant cross-species correlation on the transcriptional response to ESC differentiation. Transcription or growth factors such as GDF3, LEFTB, MYB, MYCN, NFYB, POLR3K, POU2F1, TDFG1 and UTF1 are also conserved in the transcriptional response to ESC differentiation. These conserved pathways and factors may represent fundamental molecular mechanisms regulating ESC pluripotency. These and other cross-species genomic and transcriptomic studies establish a functional portrait of ESCs [3], [8], [11]–[15].

Most studies on ESC transcriptomes focus primarily on fold changes of individual genes while overlooking concerted transcriptional changes of genes. It has been shown that modular and dynamic behaviors of gene expression are important mechanisms used by cells in functional regulation [16]–[19]. Various studies have demonstrated the significance of examining gene co-expression in addressing biological problems [20]–[24]. Cross-species analysis of transcriptional co-expression, which has never been conducted on ESCs, will facilitate the understanding of the large-scale organization and evolution of the ESC transcriptome and the molecular mechanisms of pluripotency.

In this study, we further the cross-species comparative investigation of ESCs by exploring transcriptional co-expression or modulation from pathways to global networks. We first employed generalized single value decomposition (GSVD) and comparative partition around medoids (cPAM) methods for cross-species analysis of gene co-expression in ESC-critical pathways, including ACTIVIN/NODAL, ATK/PTEN, BMP, CELL CYCLE, JAK/STAT, PI3K, TGFβ, and WNT. Complexes of co-expressed genes that were conserved across species or unique to a single species were identified, suggesting the existence of fundamental or species-specific modulation of gene expression controlling ESC pluripotency. The results suggest an essential role of JAK-mediated signaling through activating STAT2 and PI3K in ESCs, while reaffirming different requirements of STAT3-mediated LIF signaling in mouse and human ESCs. The mechanisms of WNT signaling seem to be different in human and mouse ESCs as the pathway showed differential co-expression among the key component genes across species. The AKT/PTEN pathway showed a high co-expression among the key members in both species, suggesting its fundamental role in ESC development. By promoter analysis, we identified binding sites of a set of transcription factors, including FOX, GATA, MYB, NANOG, OCT, PAX, SOX and STAT, as well the FGF response element, which may represent key regulatory mechanisms underlying the conserved co-expression in the ESC-critical pathways. By transcriptional interventions conducted *in silico*, we showed how each gene or gene combination influences pathway transitions in response to ESC differentiation or pluripotency induction. Finally, we constructed global co-expression networks of ESCs, which were dominated by a few highly-connected genes (hub genes) that link the less-connected genes to the system. The hub genes, including IGF2, JARID2, LCK, MYCN, NASP, OCT4, ORC1L, PHC1 and RUVBL1, are possibly critical in determining the fate of ESCs. Our studies demonstrate that evolutionary conservation at genomic, transcriptomic, and network levels is an effective predictor of molecular factors and mechanisms controlling ESC development. The findings and methods presented by the studies shed light on the systems understanding of how genes interact with each other to perform ESC-related functions and how ESC pluripotency or differentiation arises from the connectivity or networks of genes.

## Results

We utilized multiple microarray datasets obtained from undifferentiated ESCs and differentiated EBs of human and mouse for cross-species examination of transcriptional co-expression. Fundamental and species-specific mechanisms regulating ESC pluripotency were examined from conserved and divergent co-expression patterns in ESC-critical pathways and from transcription factors underlying the co-expression. Pathway dynamics behavior in response to ESC differentiation or pluripotency induction was determined through a series of transcriptional intervention conducted *in silico*. The global co-expression network furthermore sheds light on the overall organization of transcriptomes in ESCs.

### 1. Pathway-specific co-expression profiles

**Figure 1:**
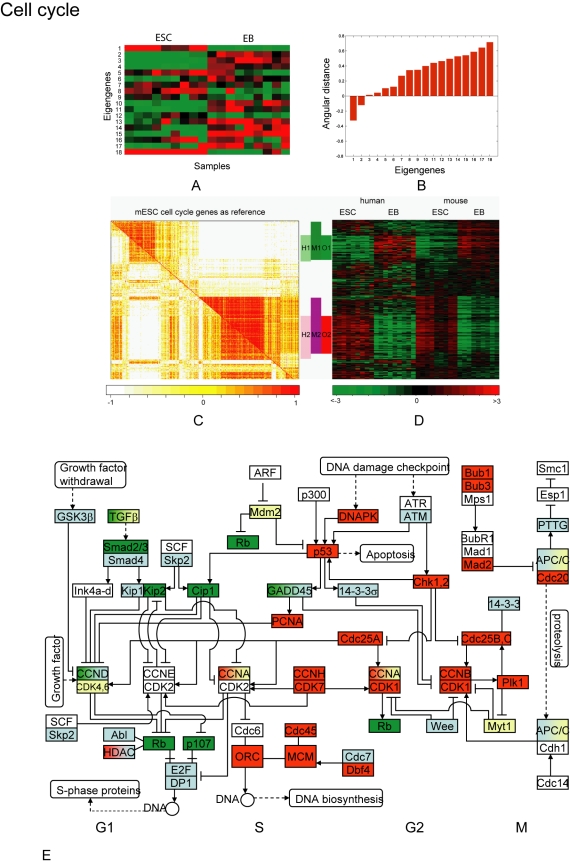
The identification of conserved and divergent co-expression gene clusters in the cell cycle from human and mouse ESC-EB data. The analysis results for AKT/PTEN, JAK/STAT, TGFβ, WNT and other pathways are presented in supplementary Figures S1 through S4. A) Heatmap display of the tailing matrix shared between human and mouse data by GSVD. The expression of each eigengene (rows) across all ESC and EB samples of both datasets (columns) is illustrated by the red-to-green color gradient representing high-to-low values. The matrix was normalized in such that all rows had the length of 1. B) Bar chart of the angular distance between singular values that shows the difference of eigengene's contribution to the total variances of human and mouse datasets. The eigengene 3 showed the smallest angular distance, indicating its almost equal contribution to the two datasets, thus used to derive conserved gene clusters across species. C) Correlation matrix of genes based on their expression profiles in human cells (the lower-left triangle part) and mouse cells (the upper-right triangle part), resulted from cPAM. The light-to-dense color gradient on the graph represents low-to-high correlation between genes. The genes are listed on the horizontal and vertical axes in the same order that was determined according to the clustering by PAM. The genes were clustered into H1 and H2 in human and M1 and M2 in mouse. The conserved co-expression cluster O1 was identified from the H1 - M1 overlapping, and the conserved co-expression cluster O2 was identified from the H2 - M2 overlapping. D) Heatmap display of the normalized expression values of the genes presented in the correlation matrix, with the genes listed in the same order. The green-to-red color gradient represents down-to-up regulation of a gene in comparison to the mean expression value across all samples. The genes in H1, M1 and O1 clusters were down-regulated, while the genes in H2, M2 and O2 were up-regulated in undifferentiated ESCs. E) Core network of the pathway with illustration of expression and co-expression patterns. The genes showing co-expression in both human and mouse cells are labeled red or green (representing up- or down-regulation in undifferentiated ESCs), the genes showing co-expression in one species but not in another are labeled blue, and the genes showing no co-expression in both human and mouse cells are labeled yellow.

By employing GSVD and cPAM algorithms, we conducted human-mouse comparative analyses on transcriptional co-expression in ACTIVIN/NODAL, AKT/PTEN, BMP, CELL CYCLE, JAK/STAT, PI3K, TGFβ and WNT pathways. These pathways are known to be critical for ESC self-renewal and differentiation [13], [25]. Taking the cell cycle as an example, we examined 356 genes of this pathway that are orthologous between human and mouse genomes and expressed in ESCs and EBs (Table S1). Figures 1-A and B illustrate the GSVD analysis. Each eigengene, computed as a linear combination of genes, represented common features between two datasets and provided a basis for identifying co-expression patterns conserved across species (Figure 1-A). Among them, the eigengene 3 showed the smallest difference between the two singular values that it was associated with (Figure 1-B), suggesting that this eigengene had nearly equal contribution to the variance of human and mouse datasets. We subsequently projected the human and mouse gene expression data onto the space of this eigengene, which led to the identification of two cross-species conserved co-expression gene clusters, C1 and C2 (Table S1). Figure 1-C illustrates the cPAM analysis, with the results summarized in <tblref rids="`, the conserved cluster O2 was identified. The conserved co-expression clusters identified by cPAM were largely similar to those by GSVD: O1 corresponded to the C1 cluster, and O2 to the C2 cluster (Table S1). By integrating together the results by the two methods, we determined the final ranges of O1 (49 genes, average *r* = 0.523 in human and 0.717 in mouse cells) and O2 (81 genes, average *r* = 0.747 and 0.851) (Table 1, details in Table S1). In total, 44% or 58% of the genes showed co-expression in human or mouse cells (*i.e.* members of clusters H1 and H2, or M1 and M2), and 37% showed conserved co-expression (*i.e.* members of clusters O1 and O2). The genes in H1, M1 and O1 were down-regulated in ESCs, while the genes in H2, M2, and O2 were up-regulated in ESCs (Figure 1-D). The co-expression in each of these gene clusters is statistically significant, beyond random expectation (P value<0.01). We finally mapped the revealed co-expression and expression patterns onto the core network of the cell cycle to further examine how the transcriptional modulation is involved in the core activities of the cell cycle in ESCs. As shown in Figure 1-E, within the core network, genes mostly showed conserved co-expression. Outside the core network, however, genes mostly showed divergent co-expression (supplementary Table S1). The analyses yielded important insights into how the cell cycle genes function through transcriptional modulation or network to control pluripotency (see details in Discussion).

**Table 1:** Summary of the identification of co-expression gene clusters in ESC-critical pathways by GSVD and cPAM. O1 and O2 are conserved clusters jointly identified by both methods.

Pathway	No of genes examined	Co-expression cluster	No of gene in cluster	Average *r*	Expression in ESCs
Cell Cycle	356	H1	60	0.473	Down
		H2	96	0.747	Up
		M1	84	0.676	Down
		M2	123	0.837	Up
		O1	49	0.523 (H)	Down
				0.717 (M)	
		O2	81	0.747 (H)	Up
				0.851 (M)	
WNT	92	H1	17	0.645	Up
		H2	19	0.59	Down
		M1	25	0.558	Up
		M2	40	0.607	Down
		O1	10	0.713 (H)	Up
				0.600 (M)	
		O2	14	0.596 (H)	Down
				0.715 (M)	
JAK/STAT	58	H1	19	0.581	Down
		H2	9	0.519	Up
		M1	22	0.658	Down
		M2	12	0.663	Up
		O1	15	0.610 (H)	Down
				0.674 (M)	
		O2	7	0.691 (H)	Up
				0.746 (M)	
TGFβ	54	H1	21	0.534	Down
		H2	3	0.861	Up
		M1	30	0.562	Down
		M2	16	0.49	Up
		O1	17	0.520 (H)	Down
				0.611 (M)	
		O2	3	0.861 (H)	Up
				0.915 (M)	
AKT/PTEN	63	H1	13	0.539	Up
		H2	18	0.601	Down
		M1	16	0.483	Up
		M2	23	0.555	Down
		O1	7	0.597 (H)	Up
				0.550 (M)	
		O2	12	0.637 (H)	Down
				0.675 (M)	

The co-expression analyses on other ESC-critical pathways were conducted in the same way as on the cell cycle. The results of GSVD and cPAM analyses, as well as the heatmaps of the identified co-expression clusters, are presented in Figures S1, S2, S3 and S4, and summarized in Table 1. As shown, two tightly co-expressed gene clusters were identified from each pathway: H1 and H2 from human cells and M1 and M2 from mouse cells. From the H1 - M1 and H2 - M2 overlappings across species, conserved co-expression clusters O1 and O2 were further identified, respectively. The identification of conserved co-expression clusters was largely consistent between GSVD and cPAM analyses. The final ranges of the conserved clusters were determined by integrating the results by both methods. The co-expression in each gene cluster was statistically significant (P value<0.001). The identification of the conserved co-expression clusters is particularly reliable as they are observed from different species. The mapping of the revealed co-expression and expression patterns on core networks of the pathways are shown in Figure S1-E through S4-E. Detailed information regarding the genes examined in each pathway, their expression patterns and classification into co-expression clusters is provided in Table S1.

### 2. Transcription factors underlying co-expression

**Figure 2:**
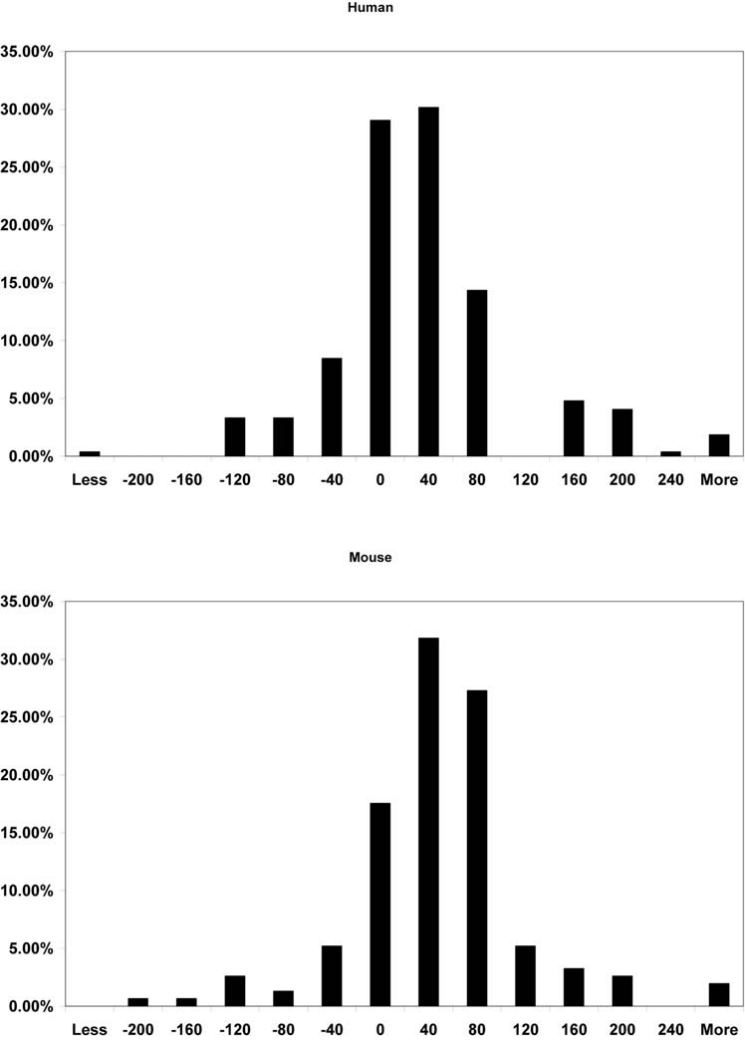
Histogram of the distances between the ETS and TCF/LEF binding sites on the promoter sequences of human and mouse genes co-expressed in ESCs. The distances were calculated based on the midpoint of binding sites from 272 (human) or 154 (mouse) proximal promoters bound by the two transcription factors. Negative or positive values on the plot indicate ETS is upstream or downstream of TCF/LEF on the promoter sequence. About 75% of the genes in the conserved co-expression clusters show an 80-bp or less distance between ETS and TCF/LEF binding sites in both human and mouse. The juxtaposed ETS and TCF/LEF binding motifs on the promoter sequences provides evidence of the FGF response element (FRE) in the conserved co-expression clusters of ESC-critical pathways.

Genes with correlated expression profiles are likely to have their promoter regions bound by common transcription factors and regulated through common regulatory mechanisms [26]. By promoter sequence analysis, we sought to uncover potential transcription factors underlying the transcriptional co-expression in the ESC-critical pathways. Table 2 lists the transcription factors that showed binding sites in most genes of both human and mouse and were statistically over-represented in each conserved co-expression cluster of the ESC-critical pathways (P value<0.01) (details in Table S2). The identification of these transcription factors should be highly reliable because it is based on the evolutionary conservation of not only genomic sequences but also transcriptomic co-expression. Among the “conserved” transcription factors, some seemed to be pathway specific as they were present only in one or two pathways (*e.g.* CART1, ETF, RUSH-1α, SF1 and SOX9), while others were present in all or most of the pathways and may represent common regulators of conserved transcriptional co-expression among different pathways. Among the latter “conserved” and “common” transcription factors, some bear no overt relationship to ESC development and may serve as new candidates for further investigation. Others have been implicated in ESC self-renewal or differentiation. For example, OCT4, SOX2 and NANOG form the central regulatory circuitry in ESCs [27], [28]. ETS and TCF/LEF, on the other hand, are effectors of ESC-critical FGF/RAS/MAPK and canonical WNT pathways [25], [29], [30]. Interestingly, the binding sites of ETS and TCF/LEF were often adjacent to each other on the promoter sequences of co-expressed genes in these pathways (Figure 2). Such juxtaposed ETS and TCF/LEF binding motifs on the promoter are referred to as the FGF response element (FRE) [31]. Being associated with most human and mouse genes in all conserved co-expression clusters of the ESC-critical pathways, the FRE appears to be evolutionary conserved and hence fundamental in regulating transcriptional modulation important to ESC development. FREs are also present on the regulatory region of SOX2 [11]. Moreover, the cognate motif of SOX2 (CA/TTTGTT) is similar to that of TCF/LEF (CTTTGA/TA/T). This raises the possibility that SOX2 competes with TCF/LEF on the FRE in cooperation with ETS proteins, or TCF/LEF competes with SOX2 on the target genes of the SOX2/OCT4/NANOG co-binding. Taken together, FGF activating the FRE may integrate with ACTIVIN/NODAL, ATK/PTEN, BMP, CELL CYCLE, JAK/STAT, PI3K, TGFβ and WNT pathways, as well as the SOX2/OCT4/NANOG regulatory circuitry, in determining the fate of ESCs.

**Table 2:** “Conserved” transcription factors which showed binding sites among most genes of both human and mouse and statistically over-represented in conserved co-expression clusters.

	Conserved transcription factors
Present in all the 5 pathways	OCT1; OCT4; CDP; CDXA; ETS; GATA4; MRF2; NCX; NKX2-5; POU3F2; SPZ1; SREBP1; SRY; TBX5; TST1; TTF1; VDR; ZF5; ZIC2; AP2; DBP; MAF; MAZ; MYB; RFX; SP3; TCF11; HAND1; E47; HNF-3ALPHA; HNF-4ALPHA; PAX; SREBP; FOX; LEF1TCF1; STAT; TEL2; NANOG
Present in only 1–2 pathways	ETF; RUSH-1ALPHA; SF1; SOX9; AR; CART1; COUP; IPF1; KROX; LXR; LYF1; MYOD; NF1; PEBP; SMAD3; SZF1-1

The transcriptional factors were identified from 5 pathways: AKT/PTEN, CELL CYCLE, JAK/STAT (incl. PI3K), TGFβ (incl. ACTIVIN/NODAL and BMP), and WNT.

More transcription factors are possible which underline the co-expression in the ESC-critical pathways, since the transcription factor database (*i.e.* Transfac) is not yet complete in the coverage of binding motif data. On the other hand, computationally identified transcription factor binding sites may not be all functionally active in cells, and ultimate experimental validation of the results is necessary.

### 3. Dynamic response of pathways to ESC differentiation and pluripotency induction

Biological pathways are dynamic and behave only in certain ways and controlled manners during development and in response to external factors [17], [22], [32]. To further explore the mechanisms controlling ESC pluripotency, we conducted a series of transcriptional interventions *in silico* on every gene or gene combination to model the dynamic behavior of pathways in transitions between ESC and EB states. In the transcription intervention, the initial expression of each gene was altered to its opposite direction (*i.e.* from initial up-regulation to down-regulation, or from down- to up-regulation) or to keep the direction the same. Three different kinds of transcriptional interventions were conducted: *a*) single-gene intervention, *b*) double-gene intervention, and *c*) triple-gene intervention (See Methods). The probabilities of network transition from the ESC to EB state and from the EB to ESC state in response to these interventions were then calculated. The genes or gene combinations showing high probabilities of the ESC-to-EB network transition were regarded as highly contributive to ESC differentiation, while genes showing high probabilities in the EB-to-ESC network transition were regarded as highly contributive to ESC self-renewal or pluripotency induction from differentiated cells. The highly contributive genes or gene combinations and their intervened transcriptional patterns provide clues for which and how experimental perturbation should be conducted for directed-differentiation of ESCs or for pluripotency maintenance or induction.

We selected JAK/STAT and WNT pathways for dynamic behavior analysis. The two pathways are critical to human and mouse ESCs, but present different intra-pathway co-expression patterns and possibly different regulatory mechanisms between species (see Discussion). The expression patterns of the genes in these pathways and the pathway topologies are shown in Table S1, Figures S1-E and S2-E.

#### JAK/STAT pathway

**Figure 3:**
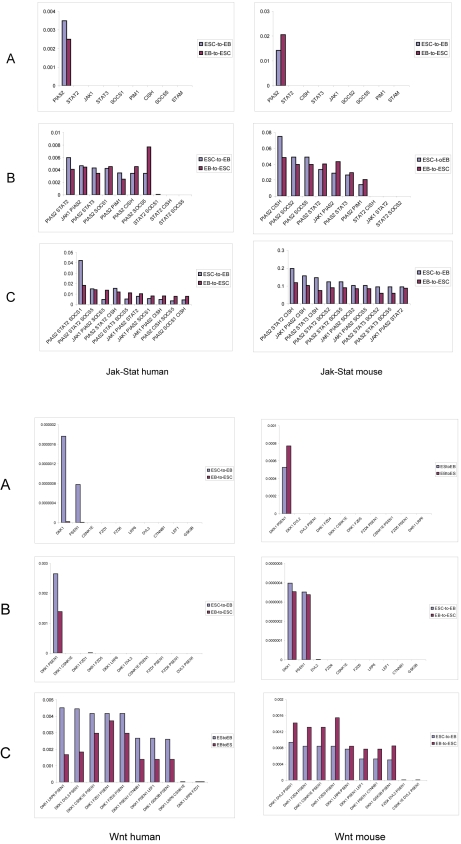
Probabilities of pathway behavior transition between ESC and EB states in response to transcriptional intervention on all individual genes (A), top 10 double-gene combinations (B), and top 10 triple-gene combinations (C) in JAK/STAT and WNT pathways of human and mouse. Each of the genes and gene combinations is listed for the maximum transition probability.

We examined the dynamic behavior based on the following key component genes of this pathway: CISH, JAK1, PIAS2, PIM1, STAM, STAT2, STAT3, SOCS2 (in mouse) or SOCS1 (in human), and SOCS5 (showing a different expression pattern from that of SOCS1 and SOCS2). The probabilities were calculated for the pathway transitions in response to 27 single-, 324 double- and 2,268 triple-gene interventions introduced on these genes. The results are shown in Figure 3 (details in Table S3). In both human and mouse cells, PIAS2 appears to be the most contributive gene to both ESC-to-EB and EB-to-ESC transitions, followed by STAT2 and JAK1 (in human) or CISH (in mouse). In other words, among all the genes, PIAS2 would the most likely cause the ESC-to-EB transition of the pathway behavior (probability 0.0035 in human and 0.014 in mouse) when its initial transcription state of up-regulation in ESCs is altered to down-regulation (Table S3). PIAS2 would also the most likely cause the EB-to-ESC transition of this pathway (probability 0.0028 in human and 0.021 in mouse) when its initial down-regulation in EBs is altered to up-regulation. Double- and triple-gene combinations in which PIAS2 was involved also showed a high transition probability in both directions when the transcription of these genes was altered. PIAS2 is an inhibitor of STAT, negatively regulating JAK/STAT signaling, along with the feedback loops of SOCS and CISH (Figure S2-E). Because of the high impact of PIAS2 on the dynamic behavior of this pathway, the knockout of this gene in ESCs may facilitate differentiation of ESCs, while increased PIAS2 production may enhance the self-renewal of ESCs or benefit induction of a differentiated cell to the pluripotent state. We furthermore found that in both human and mouse cells, the total of the transition probabilities by all single-gene interventions, as well as that by all double-gene interventions or by triple-gene interventions, was similar between the ESC-to-EB transition and the EB-to-ESC transition (Table 3). The result suggests that the JAK/STAT pathway is equally contributive to ESC differentiation (*e.g.* from ESCs to EBs) and to pluripotency maintenance or induction (*e.g.* from EBs to ESCs).

**Table 3:** Sums of probabilities of network transition between ESC and EB states under different transcriptional interventions conducted on JAK/STAT and WNT pathways.

Pathway	Species	Single-gene intervention		Double-gene intervention		Triple-gene intervention	
		ESC->EB	EB->ESC	ESC->EB	EB->ESC	ESC->EB	EB->ESC
JAK/STAT	Human	0.0035	0.0025	0.055	0.051	0.4493	0.4486
	Mouse	0.0142	0.0206	0.4378	0.4558	4.441	4.093
WNT	Human	2.559e-06	3.121e-08	0.0027	0.0014	0.0556	0.0305
	Mouse	7.573e-07	6.981e-07	0.0006	0.0008	0.010	0.016

#### WNT pathway

Totally 30 single-, 395 double-, and 3,240 triple-gene interventions were conducted on key component genes of this pathway, including CSNK1E, CTNNB1 (β-Catenin), DKK1, GSK3B, LEF1, LRP6, PSEN1, DVL2 (in mouse) or DVL3 (in human), FZD1 (in human) or FZD4 (in mouse), and FZD5 (showing a different expression pattern from that of FZD1 and FZD4). As shown in Figure 3 (details in Table S3), in both human and mouse cells, DKK1 showed the highest probability in both ESC-to-EB and EB-to-ESC transitions, followed by PSEN1, by single-gene intervention. The DKK1/PSEN1 combination showed the highest probability in transitions of both directions by double-gene intervention. The triple-gene combinations in which DKK1 and PSEN1 were involved furthermore showed high probabilities in pathway transitions on both directions. DKK1 is an inhibitor of LRP and further to the activity of the receptor FZD, while PSEN1 is an inhibitor to β-Catenin (Figure S1-E). Both DKK1 and PSEN1 were down-regulated in ESCs. Our results indicate that transcriptional changes of these two genes in either ESCs or EBs would the most likely alter the fate of the cells toward either ESC differentiation or pluripotency induction. The WNT pathway also showed some differences between human and mouse ESCs on the dynamic behavior, despite the similarity. In human cells, the probability of the ESC-to-EB transition was much higher than that of EB-to-ESC transition. In mouse cells, however, the probability was similar between the ESC-to-EB transition and the EB-to-ESC transition. For example, DKK1 and PSEN1, the most contributive genes to network transitions in both species, showed nearly 100-fold higher probabilities in the ESC-to-EB transition than the EB-to-ESC transition in human cells, but showed similar probabilities between the two transitions in mouse cells (Figure 3, details in Table S3). Moreover, the sum of all probabilities was higher in the ESC-to-EB transition than the EB-to-ESC transition in single-, double-, or triple-gene interventions for human cells, while there was little difference between the two transitions in mouse cells (Table 3). The results suggest that the WNT pathway is more contributive to ESC differentiation than pluripotency maintenance or induction in humans, while equally contributive in determining two different cell fates in mice.

The dynamical behavior of signal pathways is highly complicated; many ligands, homologous genes, alternative splice variants and regulatory factors are involved in the signaling, and different pathways further interact or cross-talk with one others, all together impacting the dynamic response to ESC differentiation or self-renewal. Our analysis, based on the key component genes and well-defined network topology to demonstrate main patterns of dynamic behavior, represents an important first step and a novel approach in systems understanding of mechanisms controlling ESC self-renewal and differentiation.

### 4. Global co-expression network of ESCs

**Figure 4:**
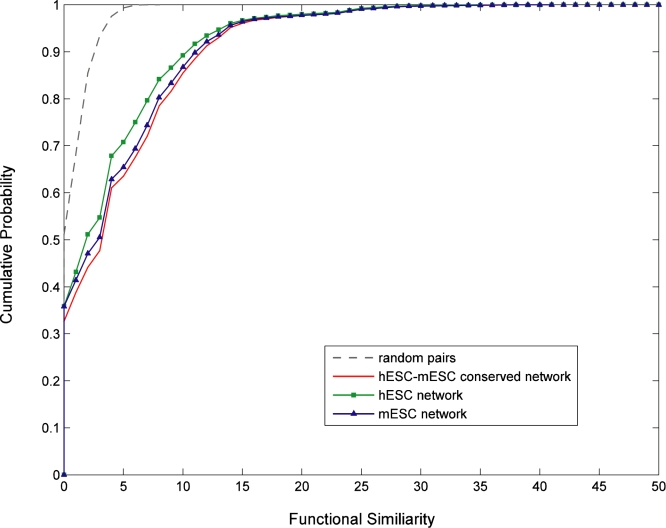
Cumulative distributions of functional similarities for co-expressed genes in ESC co-expression networks and random gene pairs. The functional similarity was calculated based on the Gene Ontology terms from *a*) 624,491 and 637,009 pairs of co-expressed genes in hESC and mESC networks, respectively (shown as the square-dotted/green, or triangle-dotted/blue solid lines, respectively, on the graph), *b*) 55,712 pairs of co-expression genes in the hESC-mESC conserved network (non-dotted/red line), and *c*) 250,000 randomly selected gene pairs (dash or grey line). For the random gene pairs, the functional similarity was 8 when the cumulative probability reached 1 (*i.e.* all genes were analyzed). For the co-expressed gene pairs in hESC, mESC, and hESC-mESC conserved networks, the functional similarity was over 30 when the cumulative probability reached 1. The cumulative distributions of functional similarities from the co-expressed genes were significantly different from that of the random gene pairs (P<10^−10^ by the Kolmogorov-Smirnov test). The co-expressed genes in the hESC-mESC conserved network exhibited a higher functional similarity than the hESC or mESC network (P<10^−10^ by the Kolmogorov-Smirnov test on the distributions).

Using genome-wide expression data of ESCs and EBs, we constructed global co-expression networks related to the early differentiation of human and mouse ESCs (hESCs, mESCs). A co-expression network consists of nodes representing genes and links representing co-expression between the genes. We calculated the correlation (*r*) between each gene according to their expression profiles, and determined the co-expression links on the network if *r* was above the threshold value. The threshold *r* value 0.80 (for hESCs) or 0.90 (for mESCs) that we chose corresponded to the linear regression model fitting index R^2^ value 0.84 or above, which suggests scale-free topology of the resulting network. The finally constructed co-expression networks of hESCs and mESCs consisted of 6,118 and 4,120 genes, respectively. Based on the overlapping or shared components between the hESC and mESC co-expression networks, we further constructed a hESC-mESC conserved co-expression network, which consisted of 55,712 genes. Similar to hESC and mESC networks, the hESC-mESC conserved network was also scale-free (R^2^ 0.75). The scale-free property, a common features among biological networks [18], [21], [33], [34], suggests that the topology of the ESC co-expression networks is dominated by a few highly-connected genes (hub genes) that link the less-connected genes to the system. The hub genes on biological networks are often vital and associated with lethal knockout phenotypes or critical biological functions [35]. It is expectable that the scale-free ESC co-expression networks are robust to the random deletion on most genes while sensitive to the targeted attack on hub genes. We furthermore found that the ESC co-expression networks were significantly associated with biological functions, through an assessment of functional similarities between co-expressed genes in the networks in comparison to random genes (Figure 4). Interestingly, the hESC-mESC conserved network showed a stronger functional association than other networks, suggesting that natural selection favors the co-expressed gene pairs that are functionally coupled over those with loose functional association in the networks. Table S4 lists the genes on the networks, along with their network connectivity (*i.e.* the number of connected genes for each gene on the network) and expression patterns in ESCs and EBs. Figure S5 shows the topology of the conserved network, with illustration of hub genes.

Tables 4-A and B list top hub genes and hub transcription factors identified from the hESC-mESC conserved co-expression network (Table S4 for details). Interestingly, all the hub genes or hub transcriptional factors showed significant changes of the expression level between ESCs and EBs (adjusted P value≤0.05). Many of the hub genes were related to the cell cycle, development, and signaling pathways important for ESC development. For example, ORC1L and ORC2L are essential for the initiation of the DNA replication. RUVBL1, evolutionarily highly conserved and essential for viability in the yeast, flies and worms [36], plays a important role in c-MYC and WNT signaling pathways [37]–[39], and in DNA repair, chromatin remodeling and apoptosis [40], [41]. NASP, an H1 histone binding protein, may regulate early events of spermatogenesis [42]. JARID2 is involved in mouse embryogenesis by showing embryogenesis-specific expression, and participates in the negative regulation of cell proliferation signaling [43], [44]. Some hub genes, such as IGF2, LCK and PHC1, have been implicated in ESC self-renewal and differentiation. PHC1, for example, is located adjacent to genes on human chromosome 12 which are hallmarks of stem cells, including CD9, GDF3, GLUT3, NANOG, SCNN1A and STELLA [45]. The target genes of PHC1 are transcriptionally repressed in ESCs to maintain pluripotency while preferentially activated during ESC differentiation [46], [47]. LCK is one of the SRC genes, which are implicated in maintaining pluripotency in mESCs [48], [49]. IGF2, an insulin growth factor, shows aberrant genomic imprinting, abnormal hypermethylation and an altered epigenetic status in ESCs [50], [51]. Among the hub transcription factors, HAND1, HMGA1, MYCN and OCT4 are known to be important for stem cell development [27], [52]–[54]. HOPX, NFYB, TFAM, TGIF1, TRIM28 and others, however, bear no overt relationship to ESCs. Such hub transcription factors or hub genes may serve as new candidates in future investigations of ESCs.

**Table 4:** Top hub genes on the hESC-mESC conserved co-expression network.

	Network connectivity	Fold-change (hESCs)	Fold-change (mESCs)
**Top 20 Genes**			
RUVBL1	428	1.347	1.16
GART	421	1.407	1.547
LCK	383	3.418	1.354
NOL11	382	1.082	1.09
PHC1	381	2.388	1.177
ORC2L	372	1.11	0.935
PPAT	369	1.345	0.997
TRAP1	367	0.942	0.687
C14orf156	367	1.023	0.888
NLE1	362	1.184	0.66
ORC1L	361	1.738	1.046
IGF2	360	−6.529	−4.548
PRIM1	360	1.422	0.613
RANBP1	359	1.043	0.854
NASP	356	1.618	1.289
NUP93	354	0.793	0.927
JARID2	352	2.106	0.979
CEBPZ	343	1.571	0.96
PRMT3	342	1.061	1.144
NHP2L1	341	0.572	0.781
**Top Transcription Factor**			
NFYB	306	1.103	0.477
TRIM28	294	0.766	1.065
TGIF1	254	0.918	0.786
HAND1	209	−5.206	−0.717
POU5F1	199	2.995	1.902
HOPX	155	−1.482	−0.558
TFAM	149	0.845	0.682
MYCN	142	0.923	1.633
FUBP1	137	0.703	0.949
HMGA1	129	0.923	0.665
JUND	114	−0.779	−0.834
MEIS2	113	−4.198	−1.174
CREB3L1	111	−0.892	−0.867
CSDA	102	0.390	1.032

Positive fold-changes indicate up-regulation of genes in undifferentiated ESCs, while negative fold-changes indicate down-regulation. The up- or down-regulation is significant in all the genes in both hESCs and mESCs (adjusted P value≤0.05 by paired t-test). Top 20 hub genes and top transcription factors (connectivity >100) are listed, respectively.

## Discussion

Our human-mouse comparative examination on ESCs identified evolutionarily conserved and divergent co-expression patterns in ESC-critical pathways, which provide insight into fundamental and specific molecular mechanisms controlling ESC pluripotency.

### Cell Cycle

The cell cycle is a critical process involved in ESC development [55], [56]. Within the core cell cycle network, the genes mostly showed conserved co-expression, belonging to either O1 or O2 clusters (Figure 1-E). In specific, cyclins of S and M phases (*i.e.* CCNA2 and CCNB2) were members of O2, up-regulated in ESCs (Table S1). Cyclins of the G1 phase (CCND2 and CCND3), the retinoblastoma protein (RB) and P107 (RBL1) were members of O1, down-regulated in ESCs. Previous studies indicate that the cell cycle in mESCs relies on constitutively active CCNA∶CDK2 and CCNE∶CDK2 complexes rather than an active INK4A/CCND∶CDK4/RB∶E2F pathway [57], [58]. Our data support these experimental observations and further suggest that these mechanisms are possibly conserved among ESCs of different species. The up-regulation of S phase cyclins and down-regulation of G1 phase cyclins highlights the fact that ESCs have a shortened G1 phase and are primed for a rapid cell proliferation [55]. We furthermore observed an up-regulation and conserved co-expression among genes for minichromosome maintenance deficient proteins (*i.e.* MCM2, MCM3, MCM4, MCM5, MCM6 and MCM7), subunits of the origin recognition complex (ORC1, ORC2, ORC3, ORC5 and ORC6), replication proteins (RPA1, RPA2 and RPA3), and a DNA replication initiation factor (CDC45), all of which were members of the O2 cluster (Table S1). These results suggest that ESCs have an elevated and tightly controlled DNA replication activity and a shortened cell cycle, a conclusion also supported by various experimental data from ESCs [55], [58], [59]. In contrast, outside of the core cell cycle network, many cell cycle related genes showed a divergent co-expression (Table S1). For example, 34 genes of the M2 cluster showed co-expression in mouse but not human cells, including cell-cycle regulation factors (CCNC, CCND1), CDC elements (CDC23, CDC37 and CDC37l1), and cell-cycle phase related factors (DP1, E2F4 and SKP2). Fifteen genes of the H2 cluster showed co-expression in human but not mouse cells, including M-phase factors BUB1, KATNB1, MKI67, NCAPD2, SMAC4I1 and WEE1. Taken together, our results suggest that tight transcriptional modulation is an essential mechanism for the core activities of the cell cycle during early differentiation of human and mouse ESCs, while the conserved core activities are regulated differentially by species-specific modulation of gene expression outside the core network.

### WNT Pathway

Canonical WNT signaling is important in maintaining pluripotency in both human and mouse ESCs [60], [61]. Our examination of this pathway showed that the conserved co-expression mostly occurred among downstream target genes, while key components of this pathway were divergent on the co-expression (Tables 1 and S1, Figure S1). In specific, among the WNT ligand genes, WNT1 and WNT5b showed co-expression in human but not mouse cells, WNT2, WNT2b and WNT6 showed co-expression in mouse but not human cells, WNT3 and WNT10b showed no co-expression in both human or mouse cells, while WNT5a was the only gene showing conserved co-expression across species (Figure S1-E). Among the signal transducing scaffold factors, DVL2 was co-expressed in mouse but not human cells, while DVL3 was co-expressed in human but not mouse cells. AXIN1 and FRAT2 showed co-expression in human but not in mouse, while GSK3b, CSNK1E and CTNNB1 showed co-expression in mouse but not in human. The detailed mechanism of WNT signaling in regulating ESC pluripotency is still not clear, based on the experimental data [60]–[63]. The inhibition of G3K3B in human and mouse ESCs [60] or PP2A in mESCs [61] indicates that the activation of the canonical WNT pathway promotes ESC self-renewal. However, direct activation of the WNT pathway using a recombinant WNT3a protein demonstrates that WNT activation promotes both self-renewal and differentiation in hESCs [62], [63]. Our pathway dynamics analysis indicated that the WNT pathway is more contributive to ESC differentiation than pluripotency maintenance or induction in human cells, while equally contributive to the two different cell fates in mouse cells. Taken together, the experimental observations and computational analyses all suggest that the WNT canonical pathway plays a complex role in human and mouse ESCs, and the functions and mechanisms of WNT signaling are not evolutionarily conserved. Our results further suggest that despite unclear roles in ESCs, WNT5a may be important to ESC development as a part of conserved modulation of gene expression in canonical WNT signaling.

### JAK/STAT and PI3K Pathways

Most of genes in the pathways showed a co-expression pattern, some of which were members of conserved co-expression clusters (Tables 1 and S1, Figure S2). As indicated in Figure S2-E, STAT3 was tightly co-expressed with JAK1 and other genes in mouse cells, but not co-expressed in human cells. The result highlights the fact that LIF signaling by STAT3 activation through the JAK/STAT cascade is required in mouse, but not in human, ESCs [64], [65]. On the other hand, JAK1, P40/ISGF3G, STAT2, certain receptors (CNTFR, GHG, IFNGR and IL10RB) and downstream target genes (BCL2L1, CCND2 and CCND3), as well as PI3K pathway elements (AKT1, PIK3R1, PIK3CD and PIK3R4), were co-expressed in both human and mouse cells as members of the conserved co-expression cluster O1 (Figure S2-E, Table S1). This observation indicates that JAK-mediated signaling through activating STAT2 and PI3K plays a fundamental role to ESC differentiation across species. Moreover, the conserved co-expression among PI3K pathway members (AKT1, PIK3CD, PIK3R1 and PIK3R4) (Table S1) suggests that transcriptional modulation in the PI3K pathway is an essential mechanism for both human and mouse ESCs. The fundamental role of the PI3K pathway in ESCs, as suggested by the computational analysis, is supported by different lines of experimental evidence. As reported, the PI3K pathway can be activated by LIF or insulin signaling and inhibits the activity of GSK3β, a WNT signaling inhibitor, in mESCs [66], [67].

### TGFβ network

The 54 orthologous genes examined for this network include members of ACTIVIN/NODAL and BMP pathways (Tables 1 and S1, Figure S3). The ACTIVIN/NODAL pathway showed a conserved co-expression pattern in human and mouse cells: key pathway components such as signal transducers SMAD2 and SMAD3, the target and repressor gene FST and the target gene PITX2 belonged to the conserved cluster O1, while the co-receptor TDGF1 and antagonist LEFTB belonged to the conserved cluster O2 (Figure S3-E). The conserved co-expression pattern suggests that tight transcriptional modulation is an essential mechanism for ACTIVIN/NODAL-directed signaling, and that the pathway is fundamental in human and mouse ESCs. Consistent with the computational analysis, experimental data from hESCs suggest a critical role of ACTIVIN/NODAL signaling for maintaining pluripotency [68]–[70]. In contrast, the BMP pathway showed a divergent co-expression pattern across species: key components of this pathway, such as receptors BMPR1A (ALK3) and BMPR2, signal transducers SMAD1, SMAD4 and SMAD5, and the target gene ID1 were co-expressed in mouse but not in human cells (Figure S3-E, Table S1). The divergent co-expression pattern suggests different mechanisms of BMP signaling in human and mouse ESCs, a result consistent with experimental data. Reportedly, in mESCs, BMP signaling through SMAD1/5 collaborates with LIF signaling to maintain pluripotency [71]. In hESCs, however, BMP signaling is suppressed and its activation induces differentiation along the trophectoderm path [72], while ACTIVIN/NODAL signaling through SMAD2/3 contributes to the maintenance of pluripotency [70].

### AKT/PTEN Pathway

Many genes of this pathway were co-expressed, including some key component genes that showed conserved co-expression (Tables 1 and S1, Figure S4). In particular, PIK3R4, phospholipase C (PLCB3 and PLCG2), the signal transducer PDK1, and the downstream target gene FOXO1 belonged to the conserved cluster up-regulated in ESCs. PIK4CA, the signal transducer AKT1, kinases PIP5K1C, TESK1 and ITPKB, phosphatases INPPL1, PLCD1 and INPP5A, and downstream target genes CCND2 and CCND3 belonged to the conserved cluster down-regulated in ESCs. Since these key pathway components, along with downstream target genes, form tight transcriptional modulation that is conserved across species, the pathway likely plays a fundamental role in regulating ESC development.

Through the cross-species analyses of co-expression from pathways to global networks, examination of transcriptional factors underlying the co-expression, and exploring the dynamic behavior of pathway via transcriptional interventions, our studies demonstrate that evolutionary conservation at genomic, transcriptomic, and network levels is an effective predictor for molecular mechanisms regulating ESC pluripotency. The computational approaches undertaken in these studies are effective in dealing with the multiple complex data to indentify major target genes and transcriptional themes, and justified by the results being frequently supported by existing experimental data. The computational studies provide a “road-map” for future experimental investigations on ESCs.

Recent breakthroughs on induced pluripotent stem cells (iPSCs) have been considered as a milestone in science and medicine. The cells are derived from non-pluripotent somatic cells, without the controversial use of embryos. iPSCs are believed to be identical to ESCs in many respects, such as the expression of signature genes and proteins, the chromatin methylation pattern, the formation of EBs, teratoma and viable chimera, and potency and differentiation ability [73]–[75]. However, the full extent of their relation to ESCs has not yet been determined. The question still remains on how to characterize “pluripotent cells” or define “stemness” for stem cells derived from different tissue sources. Our studies provide a new avenue to address this issue. The transcriptomes of stem cells can be readily examined by general organization properties of biological networks. The relationship between different stem cells can be assessed on the basis of global or pathway-specific co-expression patterns, network hub genes, transcriptional modules, and other network features. Mathematical modeling of dynamic behavior of pathways, followed by gene perturbation or knock-out experimentation, is promising in reprogramming stem cells for directed differentiation into specific cell types, or reprogramming somatic cells for pluripotency induction.

In summary, this study examined transcriptional co-expression guiding ESC self-renewal and differentiation from pathways to global networks under the framework of cross-species comparison. Conserved or divergent co-expression patterns identified from ESC-critical pathways suggest fundamental or species-specific mechanisms of transcriptional modulation important for ESC development. The promoter analysis identifying transcription factors provides evidence of co-regulation underlying the co-expression or modulation in the pathways. The mathematical modeling of pathway dynamic behavior allows the identification of genes highly contributive to network transition in response to ESC differentiation or pluripotency induction. The global co-expression networks provide an overall view of the organization of ESC transcriptomes. The hub genes identified from the network are related to important functions and possibly critical in determining the fate of ESCs. Various novel molecular mechanisms regulating ESC development are predicted, which could be further tested via independent experiments. The findings and methods presented by the studies shed light on the systems-level understanding of how genes interact with each other to perform ESC-related functions and how ESC pluripotency or differentiation arises from the connectivity or networks of genes.

## Materials and Methods

### Genome-wide expression data

We assembled a set of genome-wide gene expression profile data determined from different cell lines of human and mouse ESCs and EBs from several different sources. The human ESC and EB expression data were determined from BG01, BG02 and BG03 cell lines in our previous studies using Illumina's BeadArrays [5], [12], [76], and from H1 [3] and HES2 (E-MEXP-303 of the ArrayExpress database) cell lines using Affymetrix chips. The mouse ESC and EB expression data were determined from V6.5 (GSE3231 of GEO database), R1 (GSE2972) and J1 (GSE3749) cell lines, based on Affymetrix chips. The final data sets contained 9 ESC and 9 EB (14-day differentiated) samples from human and mouse cells, respectively. The human-mouse orthologous gene pairs were obtained from the Affymetrix probe database. The transcripts with low signal levels were removed, and the final list contained 6,573 human-mouse orthologous genes. The gene expression data were normalized using the quantile method (for the BeadArray dataset) or the RMA method (for the Affymetrix datasets). The normalized data were further converted into log2 ratios of expression values over the average expression value across all the samples for each probe. The genes differentially expressed between ESCs and EBs were identified by the paired t-test, with the P value adjusted for the false discovery rate using the Benjamini-Hochberg algorithm. The fold-change of the gene expression level was measured as the difference of mean expression levels between ESCs and EBs. Positive fold-changes indicate up-regulation of genes in undifferentiated ESCs, while negative fold-changes indicate down-regulation.

### Identification of co-expression gene clusters within pathways

GSVD and cPAM algorithms were employed to identify conserved and divergent co-expression patterns in pathways from the gene expression data of human and mouse ESCs and EBs. ATK/PTEN, CELL CYCLE, JAK/STAT (including PI3K), TGFβ (including ACTIVIN/NODAL and BMP) and WNT pathways were examined in this study. The pathway data were adopted from the KEGG database (www.genome.ad.jp/kegg) with a few modifications. Table S1 lists the orthologous genes that were examined in each pathway.

#### GSVD (Generalized Singular Value Decomposition)

Let the expression profile data of *n* genes in *p* samples (assume *n*>*p*) from two species be tabulated in matrices **M** = [**m**_1_, **m**_2_ …**m** *_n_*]^**T**^ and **H** = [**h**_1_, **h**_2_ …**h** *_n_*]^**T**^, respectively. **m** *_i_*∈ℜ^1*xp*^ and **h** *_i_*∈ℜ^1*xp*^ denote the data column vectors. GSVD is given by a pair of decompositions:
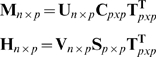
(1)where **C** and **S** are diagonal matrices with singular value elements (*c*_1_, *c*_2_ …*c_p_*) and (*s*_1_, *s*_2_ …*s_p_*), respectively, and meet 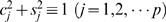. **U** and **V** are column-orthogonal matrices. The tailing matrix, **T^T^** which relates the two datasets, is invertible but not orthogonal [77], [78]. The rows of matrix **T^T^**, *i.e.* the columns of matrix **T**, **t**_1_, **t**_2_ …**t** *_p_*, list the expression of *p* latent factors, called eigengenes, across different samples in both datasets simultaneously. The relative contribution of each eigengene to each dataset is measured with the fraction of variance it captures, calculated as the ratio of the square of the corresponding diagonal element in matrix **C** (or **S**) with the sum, scaled with the length (inner product) of the corresponding eigengene vector (Eq. 2).
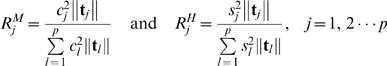
(2)In defining co-expression gene clusters, two projection matrices of the expression of *n* genes onto the *p* eigengenes are firstly generated (Eq. 3):
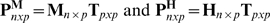
(3)The gene clusters are then identified based on the sorted projection values under each eigengene. Genes showing relatively high or low projection values under each eigengene are the most corresponding to an eigengene and are grouped together into clusters.

The conserved gene clusters are identified from the eigengene that shows a minimal difference between singular values derived from two datasets, as described in [78]. The difference between the two singular values of an eigengene is measured by the angular distance (Eq. 4):
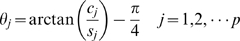
(4)An angular distance of zero indicates an eigengene has equal contribution or significance to both datasets. We first find the eigengene *j*′ which has a minimum value of *θ_j_*. We then rank genes by sorting the projection values of two data sets under eigengene *j*′ (*i.e.* the *j*′ th columns of **P^M^** and **P^H^**). We choose two clusters 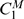 and 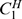 as the clusters containing 10% of all genes with large projection values of two datasets under eigengene *j*′, and two clusters 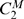 and 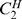 as the clusters containing 10% of genes with small projection values of two datasets under eigengene *j*′. Finally we obtain two conserved gene clusters *C*_1_ that contains common genes between 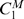 and 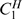, and *C*_2_ containing common genes between 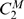 and 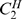.

#### Comparative partition around medoids (cPAM)

cPAM employs the partition around medoids (PAM) algorithm to perform gene clustering on two different datasets. PAM is robust to noise and outliers [79], [80]. In partitioning the dataset into *K* clusters, PAM minimizes the total intra-cluster variance, or, the squared error function 
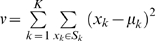
, where there are *K* clusters *S_k_*, *k* = 1,2,…, *K*, and *μ_k_* is the medoid point of all the points *x_k_*∈*S_k_* [79], [80]. The procedure starts by partitioning the input points into *K* initial sets, followed by calculating the medoid of each set. A new partition is constructed by associating each point with the closest medoid. Then the medoids are re-calculated for the new clusters, and the process repeats by alternative application of these two steps until convergence is obtained, that is the points no longer switch clusters (or alternatively medoids are no longer changed). The results of PAM on human and mouse expression datasets are then compared in a similar way as described in [23]. The procedure starts with assigning one species as the primary species, and the genes are clustered according to their expression profiles in this species. The genes of the second species are then arranged together on the matrix according to the clusters identified in the primary species. The procedure repeats by assigning the second species as the primary species.

#### Statistical evaluation of the significance of co-expression in each gene cluster

The statistical significance of co-expression of each gene cluster was assessed by a random scrambling test. For a gene cluster with *n* genes, Pearson's correlation coefficients were calculated for all gene pairs across all samples. The average correlation coefficient (average *r*) of the gene cluster was then compared with average *r* values of 10,000 gene sets generated by randomly choosing *n* genes from all the genes in the dataset. The frequency of having random average *r* values greater or equal to the observed average *r* value was taken as the P value of observing the level of co-expression in the gene cluster with *n* genes.

### Identification of transcription factor binding sites from promoters

The 7 kb proximal promoter sequences of genes in each conserved co-expression cluster were retrieved from the Promoser database (biowulf.bu.edu/zlab). Transcription factor binding sites were identified by searching the promoter sequences against the vertebrate matrix data set of the Transfac 9.0 database, using the software Match (www.gene-regulation.com). The cutoff matrix similarity was set to 0.8 and core similarity to 0.85 in the search. The binding sites of the transcription factor NANOG were detected based on the consensus binding sequence (C/G)(G/A)(C/G)C(G/C)ATTAN(G/C) [81]. The FRE was detected by scanning ETS binding motifs (EBM) and TCF/LEF binding motifs (TLBM), which lie adjacent to the same strand of the promoter sequence. The EBM was recognized based on the consensus binding sequence A/CGGAA/T [82] and the TLBM was based on CTTTGA/TA/T [83]. The transcription factors or FREs that show binding sites in more than 50% of human genes and mouse genes and are statistically over-represented (P value<0.01 by Fisher's exact test) among the genes of a cluster are considered as significant ones underlying co-expression of that gene cluster.

### Analysis of pathway dynamic behavior

We developed an algorithm which is based on a finite-state Markov chain model to mimic the dynamic change of a network in response to a series of transcription interventions made *in silico* on each gene or gene combination in the network [32]. The inputs to the analysis algorithm are pathway topology and gene expression data. The outputs are the estimated probabilities of network transition between two states (*i.e.* ESCs and EBs). Prior to the mathematical simulation, real-value gene expression data are converted to the ternary presentation, so that each gene is assigned as either over-expressed (1), equivalently-expressed (0), or under-expressed (−1). This is to ensure a high and uniform certainty in specifying genes undergoing significant transcriptional changes across different conditions.

For capturing the dynamics of the network with *n* selected genes, we used the state of predictor genes at step *t* and the corresponding conditional probabilities, which are estimated from observed data, to derive the state of target gene at step *t*+1, as characterized by Eq. 5.
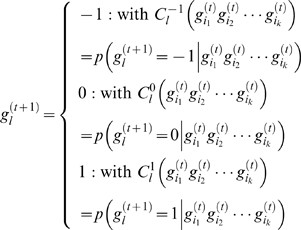
(5)where *i*_1_, *i*_2_, …*i_k_*, *l*∈{1, 2, …, *n*} and *k* is the number of predictor genes. 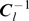, 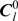, and 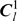 are conditional probabilities that depend on the states of the predictor genes and satisfy 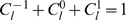. The transition between gene states can be represented as a Markov chain. Considering gene perturbation, the transition probability can be formulated by Eq. 6.
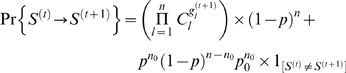
(6)where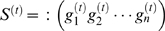, *p* is the perturbation probability for each gene, 
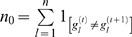
 is the number of genes to be perturbed, and *p*_0_ = 1/(*q*−1). In ternary case, *q* = 3, so *p*_0_ is equal to 0.5.

Based on the transition matrix of the model, we constructed the intervention information matrix **H**, in which the element is defined as 
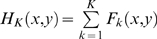
, where *F_k_*(*x*, *y*) can be computed recursively as 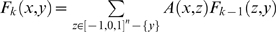 for *k*≥2 and *F*_1_(*x*, *y*) = *A*(*x*, *y*) for *k* = 1. Each element *A*(*x*, *y*) of the transition matrix **A** can be computed using Eq. 6. Each column in **H** represents the probability that the network, will visit to a given target state *y*, starting in all possible intervened states. Using the intervention information matrix **H**, we can find the best candidate genes that we intervene with for the highest transition probability. We then conduct transcription intervention *in silico*. Three different kinds of transcriptional interventions were conducted in this study: *a*) single-gene intervention, in which a single gene was intervened each time, across all genes, *b*) double-gene intervention, in which two genes were intervened simultaneously each time, across all two-gene combinations, and *c*) triple-gene intervention, in which three genes were intervened simultaneously, across all three-gene combinations.

### Analysis of global co-expression networks

Human-mouse orthologous genes were used for constructing global co-expression networks of human and mouse ESCs, respectively. The Pearson correlation coefficient value (*r*) was first calculated based on the expression profiles for each gene pair. The co-expression links in the network were kept if the corresponding *r* values are at or above a threshold *T_r_*. The value of *T_r_* is determined according to the scale-free criterion, which is measured by the square of the correlation coefficient (R^2^) between log(P(*k*)) and log(*k*), where *k* denotes the connectivity of a node, or the number of links of a node to other nodes in a network. P(*k*) gives the probability that a selected node has exactly *k* links, which is calculated as the number of the nodes (genes) at a given *k* value divided by the total number of nodes. The *T_r_* values 0.80 and 0.90 that we determined from human and mouse data corresponded to R^2^ values 0.88 and 0.84, respectively. At or above the threshold values, genes were considered to be co-expressed and the derived networks obeyed a power law distribution and were scale-free. Such a scale-free criterion removed possible spurious co-expression links and so that the resulting networks are biologically meaningful.

The functional relevance of co-expression networks was assessed based on the Gene Ontology (GO), in which each gene is described by a set of GO terms. The level 3 biological process terms in the GO database were used for this analysis. The functional similarity of a pair of genes A and B is measured by the number of GO terms that they share, (GO_A_∩GO_B_), where GO_A_ or GO_B_ denotes the set of GO terms for genes A or B. The functional similarity is set to zero if one or both genes have no terms. The functional similarity was calculated from co-expressed gene pairs in the co-expression networks, as well randomly selected gene pairs. The resulting cumulative distributions of functional similarity scores of genes being analyzed are examined by the Kolmogorov-Smirnov test for the statistical difference.

## Supporting Information

Figure S1 The identification of conserved and divergent co-expression gene clusters from human and mouse ESC-EB data for the WNT pathway. The figure legends are the same as for Figure 1.
(1.75 MB TIF)

Figure S2 The identification of conserved and divergent co-expression gene clusters from human and mouse ESC-EB data for the JAK/STAT pathway (incl. PI3K pathway). The figure legends are the same as for Figure 1.
(1.51 MB TIF)

Figure S3 The identification of conserved and divergent co-expression gene clusters from human and mouse ESC-EB data for the TGF-beta network (incl. ACTIVIN/NODAL and BMP pathways). The figure legends are the same as for Figure 1.
(1.41 MB TIF)

Figure S4 The identification of conserved and divergent co-expression gene clusters from human and mouse ESC-EB data for the AKT/PTEN pathway. The figure legends are the same as for Figure 1.
(2.08 MB TIF)

Figure S5 Topology of the hESC-mESC conserved co-expression network, with illustration of hub genes.
(2.92 MB TIF)

Table S1 Lists of examined genes in AKT/PTEN, CELL CYCLE, JAK/STAT (incl. PI3K), TGF-beta (incl. ACTIVIN/NODAL and BMP) and WNT pathways, along with their expression patterns in ESCs in comparison to EBs and classification into conserved and divergent co-expression gene clusters identified by GSVD and cPAM.
(0.15 MB XLS)

Table S2 Conserved transcription factors which showed binding sites among most genes of both human and mouse and statistically over-represented in a conserved co-expression cluster, and the number of pathways where the conserved transcription factor were present in at least one conserved co-expression cluster.
(0.03 MB XLS)

Table S3 Probabilities of network transition between ESC and EB states in JAK/STAT and WNT pathways under single-, double-, and triple-gene interventions. Transcriptional intervention is presented as: transcriptional pattern at the initial state (e.g. ESC state) = >transcription pattern after intervention = >transcription pattern at the end state (e.g. EB state). In each state, the transcription pattern of each gene are presented by ternary values (i.e. 1, −1, 0, representing up-regulation, down-regulation, and no change).
(0.70 MB XLS)

Table S4 Genes and transcription factors on the hESC, mESC, and hESC-mESC conserved co-expression networks, along with their expression patterns in ESCs and network connectivity (listed from the highest to lowest).
(1.43 MB XLS)
