# Supplementary figures and images for: Evolutionarily Conserved Transcriptional Co-Expression Guiding Embryonic Stem Cell Differentiation
Source: PLoS One. 2008 Oct 15;3(10):e3406. doi: 10.1371/journal.pone.0003406 (PMC2566604; doi:10.1371/journal.pone.0003406)

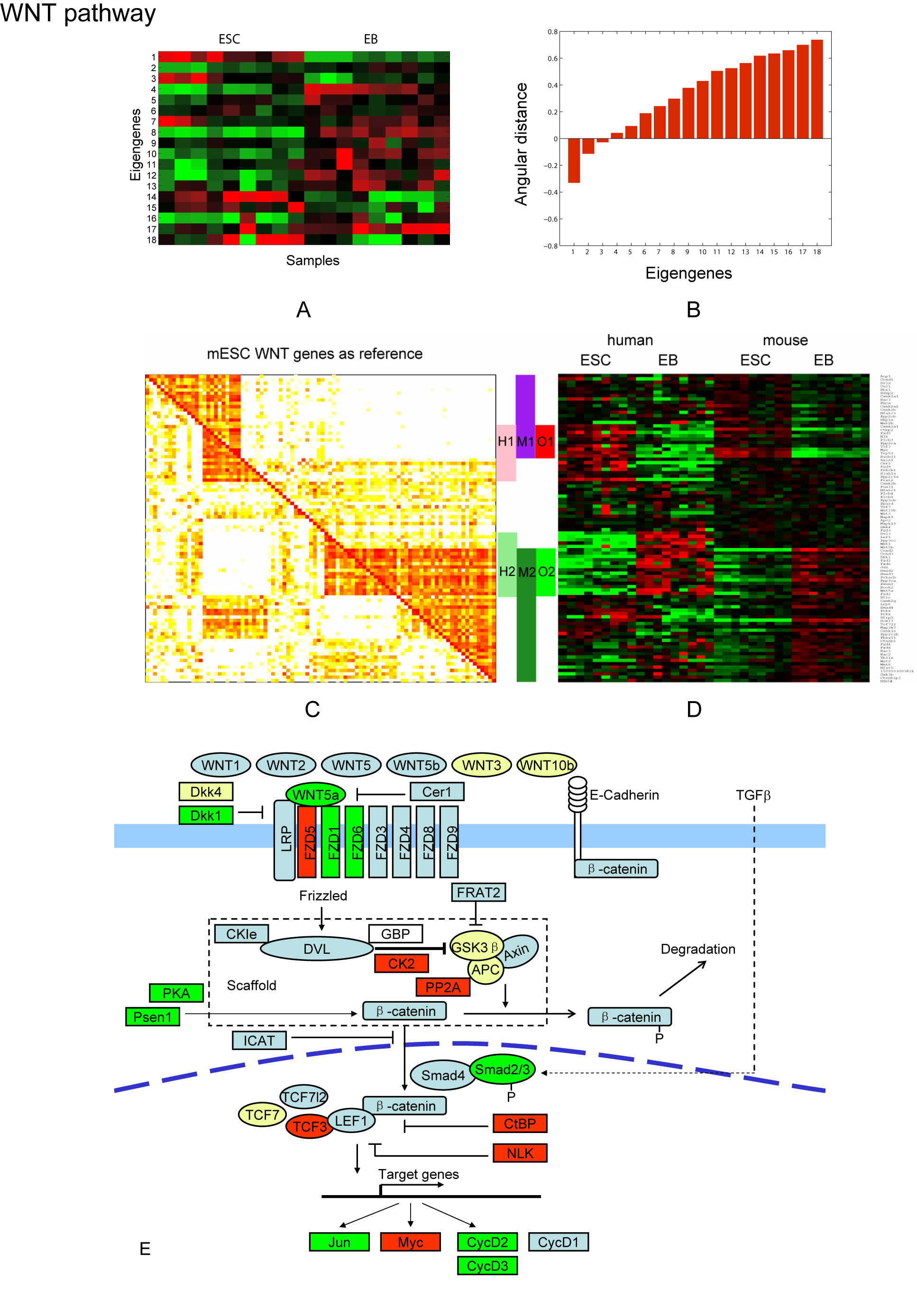

Supplement: Figure S1 — The identification of conserved and divergent co-expression gene clusters from human and mouse ESC-EB data for the WNT pathway. The figure legends are the same as for Figure 1. (1.75 MB TIF) [file pone.0003406.s001.tif]

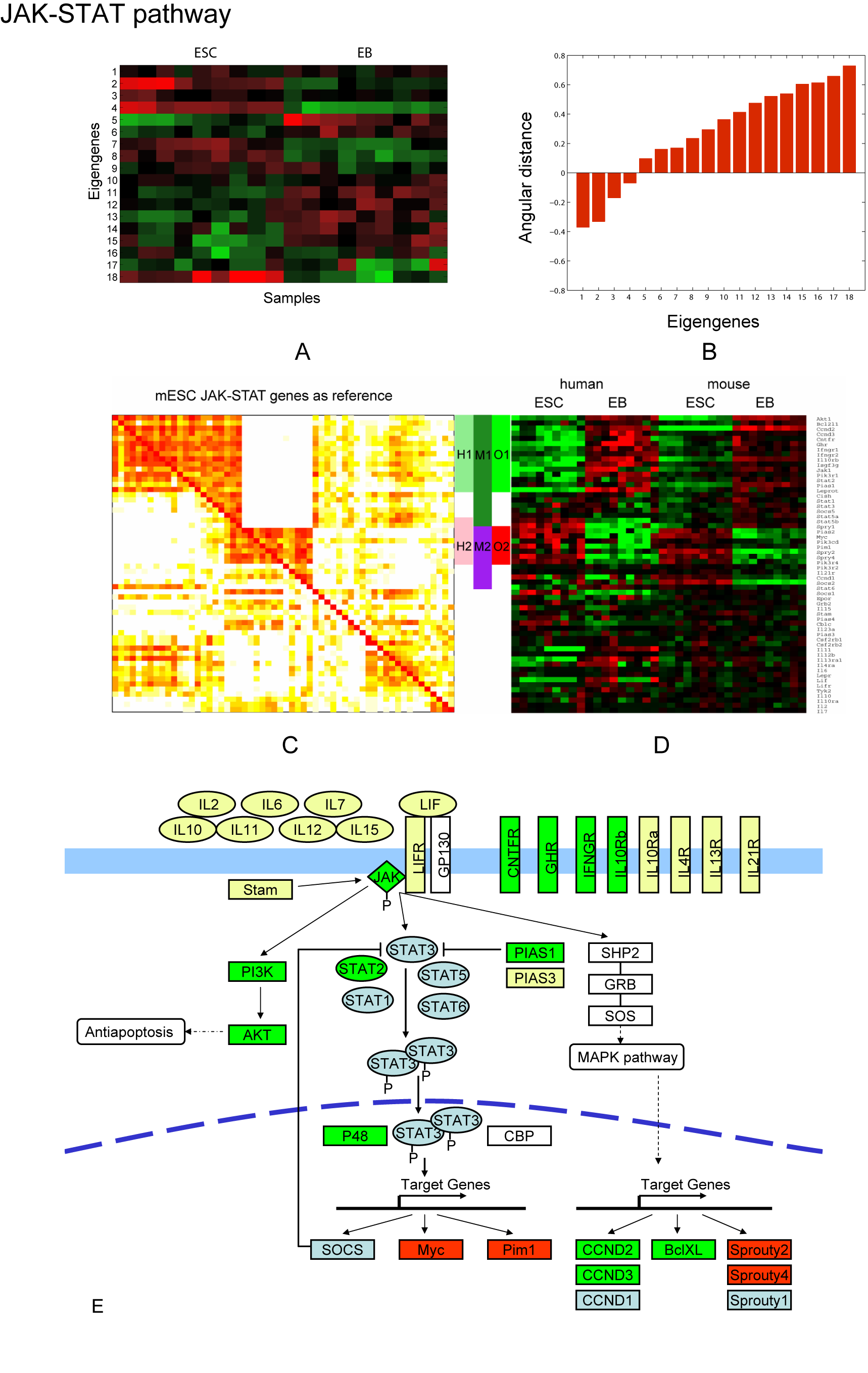

Supplement: Figure S2 — The identification of conserved and divergent co-expression gene clusters from human and mouse ESC-EB data for the JAK/STAT pathway (incl. PI3K pathway). The figure legends are the same as for Figure 1. (1.51 MB TIF) [file pone.0003406.s002.tif]

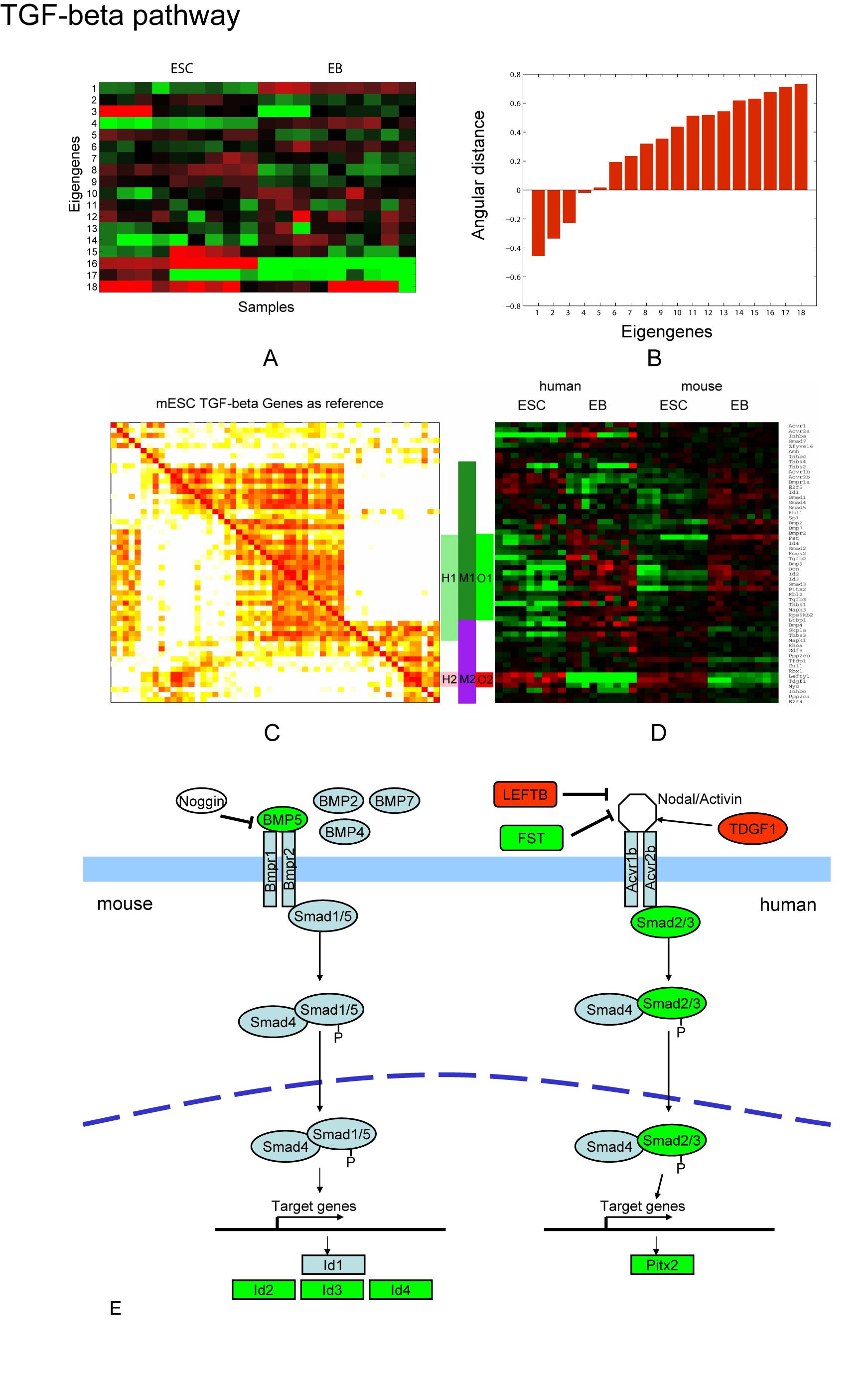

Supplement: Figure S3 — The identification of conserved and divergent co-expression gene clusters from human and mouse ESC-EB data for the TGF-beta network (incl. ACTIVIN/NODAL and BMP pathways). The figure legends are the same as for Figure 1. (1.41 MB TIF) [file pone.0003406.s003.tif]

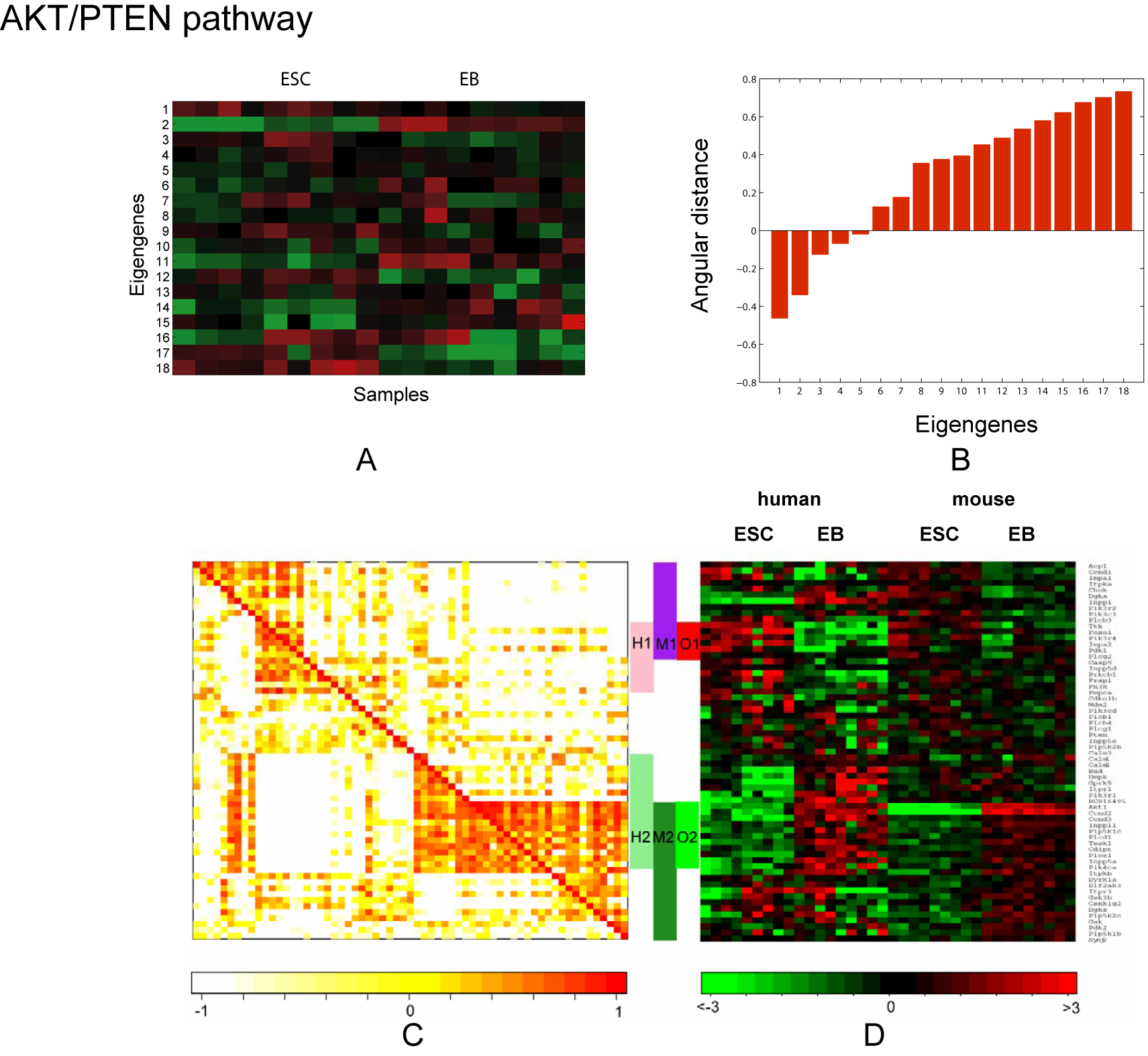

Supplement: Figure S4 — The identification of conserved and divergent co-expression gene clusters from human and mouse ESC-EB data for the AKT/PTEN pathway. The figure legends are the same as for Figure 1. (2.08 MB TIF) [file pone.0003406.s004.tif]

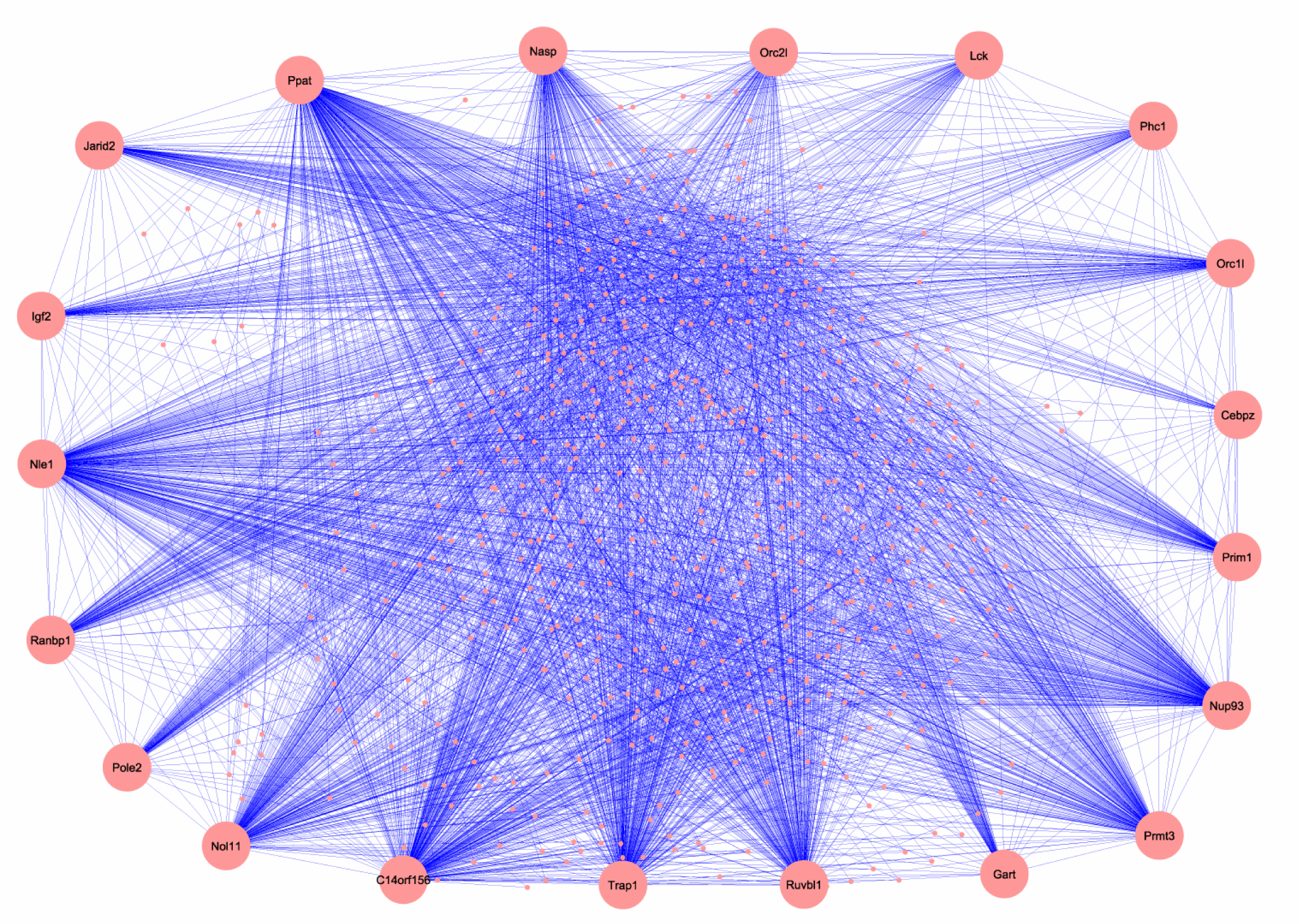

Supplement: Figure S5 — Topology of the hESC-mESC conserved co-expression network, with illustration of hub genes. (2.92 MB TIF) [file pone.0003406.s005.tif]
